# The use of canid tooth marks on bone for the identification of livestock predation

**DOI:** 10.1038/s41598-019-52807-0

**Published:** 2019-11-08

**Authors:** José Yravedra, Miguel Ángel Maté-González, Lloyd A. Courtenay, Diego González-Aguilera, Maximiliano Fernández Fernández

**Affiliations:** 1 Department of Prehistory, Ancien History and Archaeology, Complutense University, Prof. Aranguren s/n, 28040 Madrid, Spain; 2 C.A.I. Arqueometría, Complutense University, Prof. Aranguren s/n, 28040, Madrid, Spain; 3 Department of Cartographic and Land Engineering, Higher Polytechnic School of Avila, University of Salamanca, Hornos Caleros 50, 05003 Avila, Spain; 4 Gran Duque de Alba Institution, Diputación Provincial de Ávila, Paseo Dos de Mayo, 8, 05001 Ávila, Spain; 5 Department of Prehistory, Universitat Rovira i Virgili (URV), Avinguda de Catalunya 35, 43002 Tarragona, Spain; 6 Institut de Paleoecologia Humana i Evolució Social (IPHES). Zona educacional, Campus Sescelades URV (Edifici W3) E3, 43700 Tarragona, Spain; 7 Department Sciences of Communication and Sociology, Faculty of Communication Sciences, University Rey Juan Carlos, Camino del Molino, s/n, 28943 Fuenlabrada, Madrid Spain

**Keywords:** Animal behaviour, Behavioural ecology, Palaeontology

## Abstract

Historically wolves and humans have had a conflictive relationship which has driven the wolf to extinction in some areas across Northern America and Europe. The last decades have seen a rise of multiple government programs to protect wolf populations. Nevertheless, these programs have been controversial in rural areas, product of the predation of livestock by carnivores. As a response to such issues, governments have presented large scale economic plans to compensate the respected owners. The current issue lies in the lack of reliable techniques that can be used to detect the predator responsible for livestock predation. This has led to complications when obtaining subsidies, creating conflict between landowners and government officials. The objectives of this study therefore are to provide a new alternative approach to differentiating between tooth marks of different predators responsible for livestock predation. Here we present the use of geometric morphometrics and Machine Learning algorithms to discern between different carnivores through in depth analysis of the tooth marks they leave on bone. These results present high classification rates with up to 100% accuracy in some cases, successfully differentiating between wolves, dogs and fox tooth marks.

## Introduction

One of the oldest common proverbs refers to domestic canids as “man’s best friend”, yet in historic times wolves and humans have been seen to have a somewhat special relationship. Throughout the majority of the Pleistocene these two species remained indifferent to each other, while in other periods a love-hate relationship has slowly emerged^1^. From one perspective, the cooperation of wolves allowed for the development of hunting strategies in colder Eurasian ecosystems^2^, however, especially since Neolithic periods, humans and wolves have expressed a complex^1,3,4^ and sometimes conflictive^5–7^ relationship that has led to the near extinction of wolf populations in some geographic regions^8–11^.

Since the end of the 20^th^ century different conservational programs have been proposed to save these wild canid populations in many areas^5,9,10,12–14^, however, rural communities have frequently protested against the reintroduction of these predators in some areas, frequently leading to conflict^3,6,7,14,15^. Such issues are fruit of the predatory habits of wolves where, while these animals prefer to hunt wild species^15–19^, they also have a large impact on livestock, creating issues for stock breeders and farmers^5,6,8,12,13^. This results in a significant exposure of these issues within the media that has been seen to cause problems and conflict within these affected areas (Supplementary Note S1).

In response, governments have set up economic subsidies for affected landowners^3,8,16,20–26^, trying to meet a middle grounds that also favours the protection of wolf populations. This process, however, has not been free of additional social conflict (Supplementary Note S2). The root of these problems in many cases lies in delays produced in the payment of these subsidies^24^, alongside a lack of diagnostic data that can be used to reliably determine the predator responsible for domestic animal slaughter^27^. In some cases, this has even led to fraudulent claims for compensation^26^.

Detecting the predator responsible for killing an animal is often difficult^27–30^, considering how wild dogs, alongside other carnivores such as the bear, cougar or fox, are all able to hunt livestock^27,30,31^, studies have revealed populations of both wild and domestic dogs to have an important impact on an ecosystem^27,32,33^ (Supplementary Note S1), affecting all types of different animals. While some studies have tried to provide observations that distinguish between different carnivores responsible for the killing of livestock^27,34–36^, the problem with these types of variables is that they are considerably affected by the decay or organic material, leaving only skeletal remains. This is a frequent problem when other agents have intervened, such as vultures, hawks or other types of scavengers. This is generally problematic for herders and farmers when trying to reclaim compensation for their loss of livestock, increasing the tension between the affected and government officials^8–10^ (Supplementary Note S1). In some cases, studies allude the differentiation between wolves and dogs to be impossible^28,29^, suggesting genetic studies to be the only means of withdrawing conclusions from animal carcasses^27,30^.

**Figure 1:**
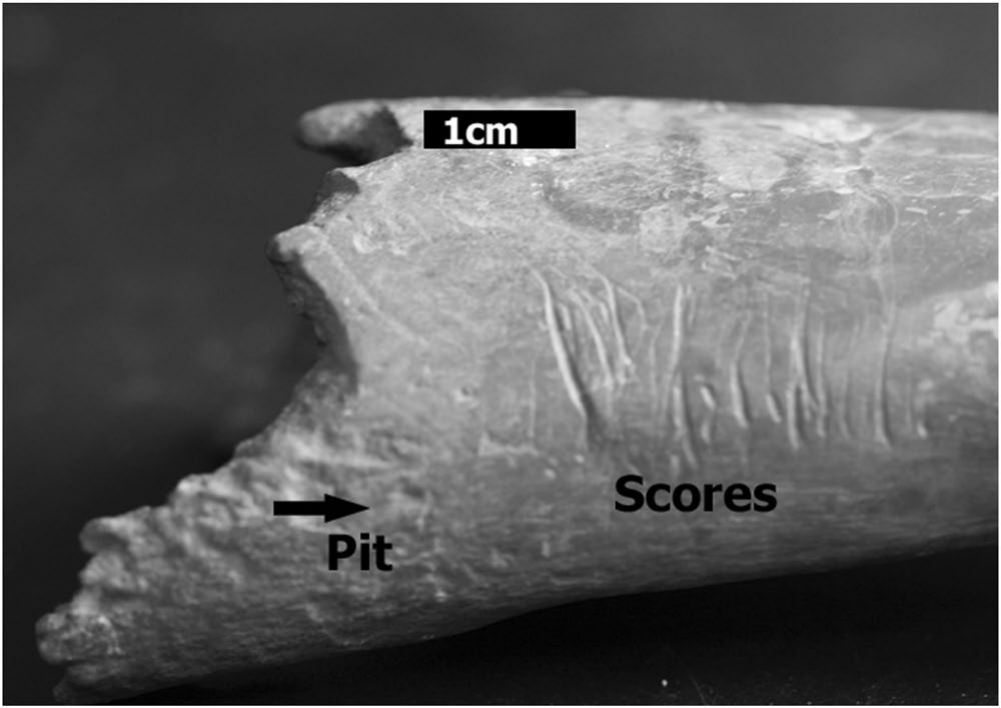
Types of Tooth Mark. Example of tooth pits and scores produced by wolves.

This study presents a new methodological approach using geometric morphometrics and machine learning to differentiate between different carnivore attacks, presenting an alternative to bite mark location based methods that can only be carried out where flesh and skin are preserved. Here we describe a new means of analyzing these types of cases, based on the analysis of the morphology of tooth marks left by carnivores on the shafts of long bones. The two most common types of tooth marks produced during carnivore feeding are known as pits, which are circular depressions, and scores, which are grooves with a length twice as long as their width, with a “U” shaped cross section^37^ (Fig. 1). The case study includes those marks produced by different predators such as wolves, dogs and foxes. The objectives to this approach are to find a means of solving the aforementioned issues, providing diagnostic criteria that can be used to distinguish some of the carnivores that are frequently considered the cause of domestic animal death.

## Results

**Figure 2:**
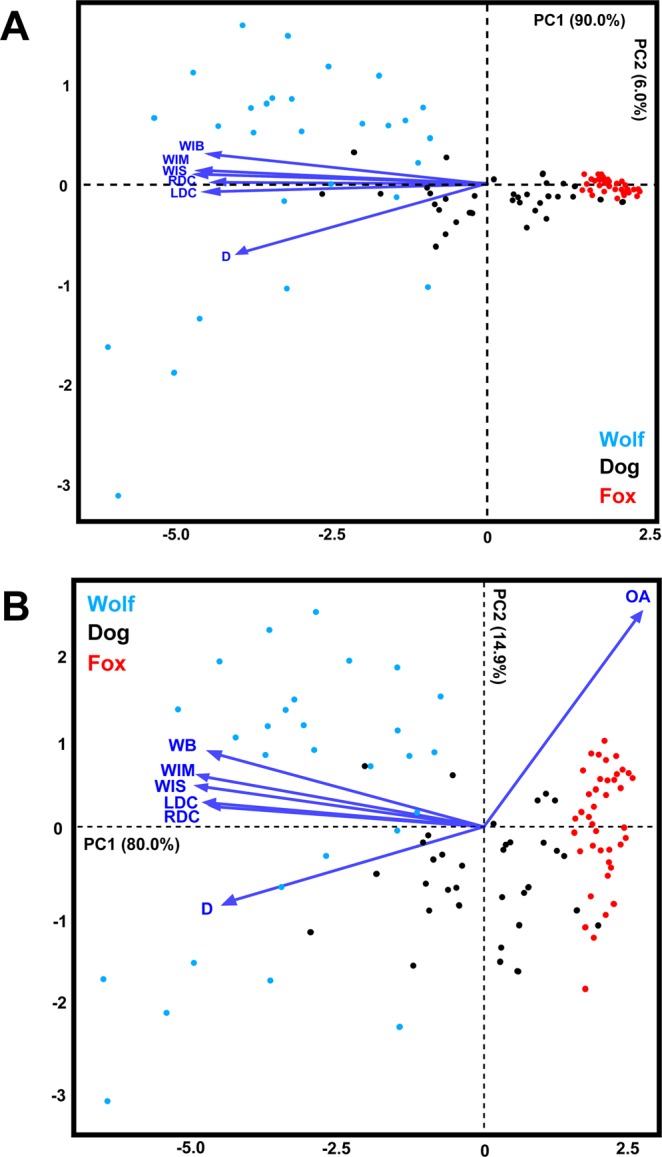
Principal Component Analysis Scatter Bi-Plots from Measurements. PCA bi-plots presenting variance in tooth score dimensions (**B**) excluding as well as including (**A**) including the variable OA.

**Figure 3:**
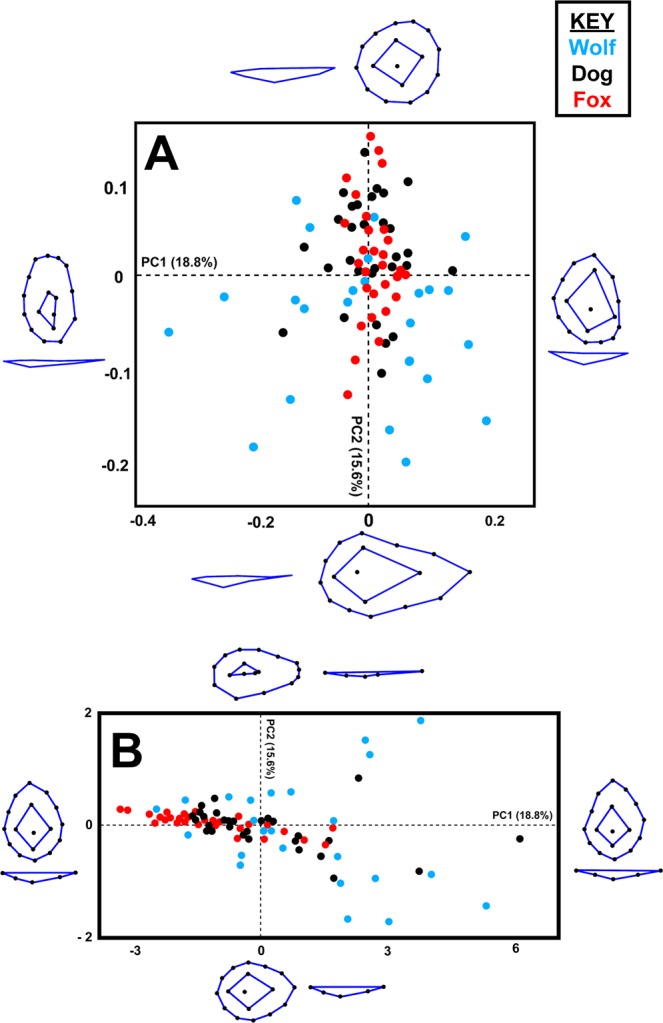
Principal Component Analysis Scatter Plots from Geometric Morphometric 2D Data. PCA plots presenting variance in tooth score cross-section morphology using the 7-landmark 2D model. Variance in shape is presented for the extremities of each PC score. (**A**) Shape space. (**B**) Form space.

**Figure 4:**
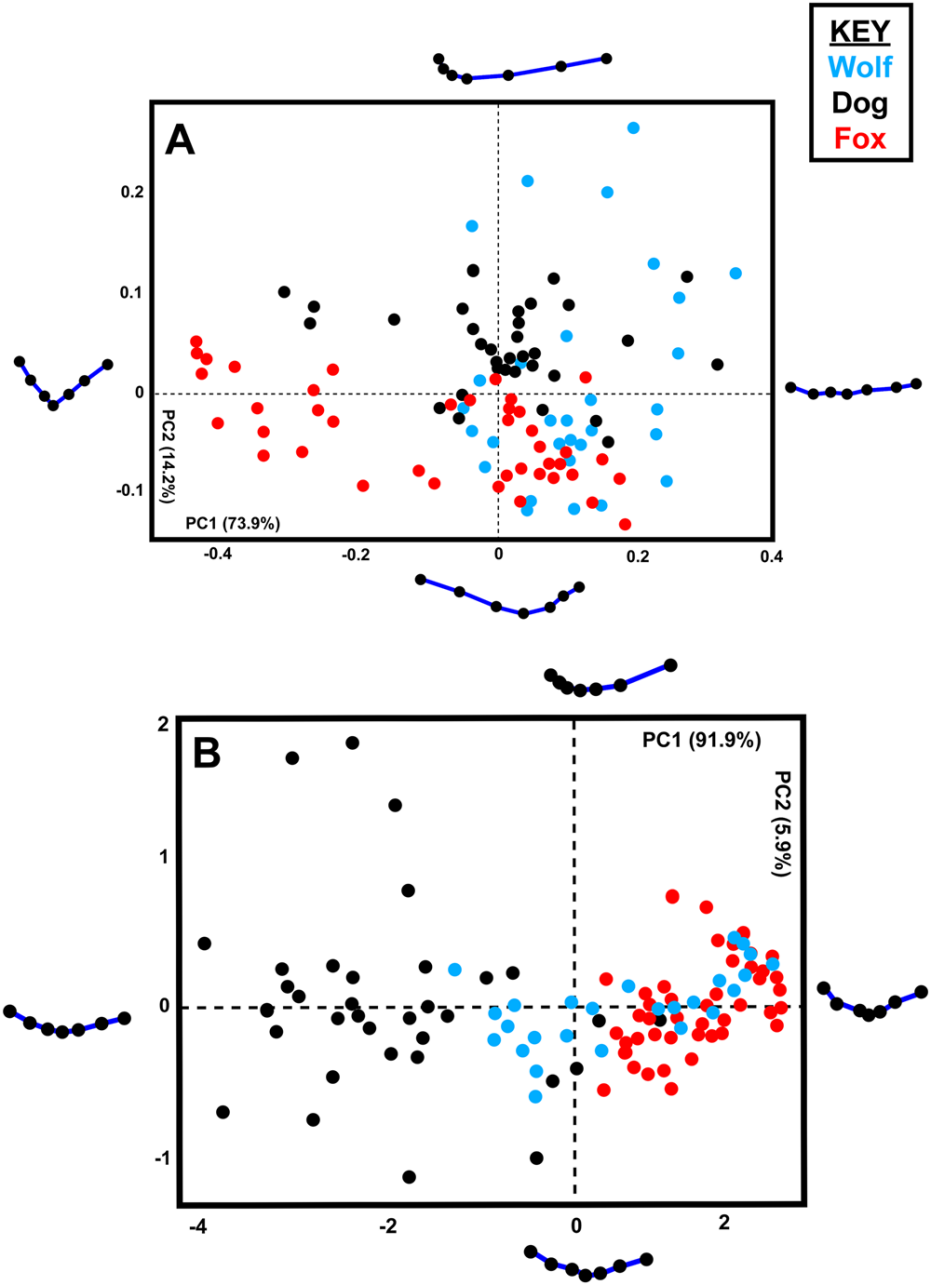
Principal Component Analysis Scatter Plots from Geometric Morphometric 3D Data. PCA plots presenting variance in tooth pit morphology using the 17-landmark 3D model. Variance in shape is presented for the extremities of each PC score. (**A**) Shape space. (**B**) Form space.

Most graphic results present a relatively high degree of separation among groups, especially in the case of biometric data (Fig. 2). In all cases the degree of separation is significant, as established by multivariate statistical results presented in Table 1. In depth evaluation of sample distribution in each principal component feature space indicate wolves to be the most variable when analyzing tooth mark morphologies. Additionally, foxes appear to be present the least amount of variation, appearing as a tight cluster in both metric (Fig. 2) and geometric morphometric PCAs (Figs 3 and 4).

**Table 1:** Multivariate Analyses comparing different carnivore tooth marks.

	Dog					
	With OA	Without OA	Score Shape	Score Form	Pit Shape	Pit Form
Wolf	0.001	0.001	0.001	0.001	0.002	0.007
Fox	0.001	0.001	0.001	0.001	0.027	0.004

Multivariate Analysis of Variance (MANOVA) *p* values comparing dog tooth marks with wolves and foxes. Results include analysis of metric variables derived from tooth score cross sections (including as well as excluding the variable Opening Angle (OA)), as well as the geometric morphometric variations in morphology of tooth score cross sections and entire tooth pit morphologies in both form and shape space.

PCAs for metric analyses are generally presented by PC scores representing a high degree of variance across the first two principal components. In both the inclusion (7 PC Scores in total) and exclusion of OA (6 PC Scores in total), the cumulative proportion of variance represented is over 90%, with the biplot revealing a tendency for PC1 to represent almost all of the variables with the exception of OA (Fig. 2). The tendency for wolves to occupy an area of the graph with larger tooth marks and the fox to appear on the opposing extremity can be logically explained considering the size of the animals being compared. Considering the much smaller dimensions of fox cuspids in comparison with those of wolves, it is reasonable to assume that the variable size holds a significant weight on the comparison of these different samples.

When observing differences in pure morphological feature space, excluding the influence that tooth mark size may have on the sample, differences are still relatively clear (Table 1). Overlapping of samples increase however patterns in feature space can still be observed for both tooth pit and score marks. Fox tooth scores remain to occupy a more restricted proportion of feature space, while wolves are represented by the highest degree of morphological variability. In shape space (Figs 3A and 4A), dog tooth marks begin to overlap more with samples produced by wolves, yet their differences to fox tooth marks remain clear. In form space (Figs 3B and 4B), overlapping remains however begins to present a clearer separation amongst samples in the case of tooth scores (Fig. 3B). In the case of scores (Fig. 3), geometric morphometric data is represented by a relatively small number of dimensions (Shape = 10 PC scores, Form = 14 PC scores) with a slightly clearer separation amongst samples than in the case of pits (Fig. 4). Nevertheless, geometric morphometric data for the case of pits is represented by a high number of dimensions (Shape = 44 PC scores, Form = 51 PC scores), and remain to present significant differences in the distributions of samples as demonstrated by MANOVA results for both shape and form (Table 1).

Exploring morphological variation through results obtained in grid warping calculations reveal foxes to produce deeper tooth marks of both types, while wolves tend to produce more superficial marks. Dogs in each study appear to occupy a midway point between both fox and wolf samples.

Finally, SVM models were able to efficiently construct hyperplanes that separated all samples with a 100% classification rate on all types of data sets (Table 2). The global loss across each model indicate highly accurate decision boundaries, trained and computed in less than 1/10th of a second. Furthermore, obtaining optimal hyperparameters in model tuning took an average of 124.9 milliseconds. Based on classification-misclassification ratios obtained in model evaluation, Kappa, Sensitivity and Specificity metrics are able to highlight the potential these models have for processing data with low rates of misclassification. Considering each of these evaluation metrics and the computational power required to run these algorithms, SVMs can be seen to be the most efficient classification models for morphological data of any type.

**Table 2:** Machine Learning Evaluation Metric Data.

	Measurements		Geometric Morphometrics	
	With OA	Without OA	Scores	Pits
Optimal Cost	13.15	222.78	22.34	22.34
Optimal Gamma	302.84	48.35	161.42	161.41
Kappa	1	1	1	1
Accuracy	1	1	1	1
Lower CI	0.99	0.99	0.99	0.99
Upper CI	1	1	1	1
MSE	7.1e-05	7.2e-05	6.7e-05	7.2e-05
Sensitivity	1	1	1	1
Specificity	1	1	1	1
Training Time (ms)	51.40	61.73	89.56	89.79

Support Vector Machine performance metrics, describing the final optimal hyperparameters used to obtain our results, all evaluation metrics as well as the upper and lower 95% confidence interval bounds for balanced accuracy values, Mean Squared Error results and the time it took to train each model.

## Discussion and Conclusions

This study presents an additional case of high classification amongst carnivore tooth marks using a hybrid geometric morphometric and machine learning methodological approach. Here we have been able to obtain 100% classification between tooth marks produced by Irish Setter gundogs, wolves and foxes. These results confirm that differentiation is possible and could provide a useful tool for discerning the agents responsible for livestock predation. This is an important advance that could be used to resolve a number of cases, such as insurance fraud^26^, and thus lower tension between landowners and government officials^23–25^.

Across areas of Europe and Northern America, the conflict between wolves and farmers is intense. To relieve said tensions, governments have directed large amounts of funding towards the compensation of livestock owners^3,8,16,20–26^, nevertheless, for many these subsidies are considered inefficient and thus push for a general reduction of wolf populations^3,24,26^ (Supplementary Note S1). This is especially dangerous in areas such as Sweden and Norway where wolf populations are especially low^20–22^. On the other hand livestock owners are currently pushing for faster and larger subvention allocations^24,26^, insisting that governments help establish plans to protect livestock including an increase in the rearing of shepherd dogs and the erection of electric fences among others^3,5–7,12,15,25,26^. Nevertheless, multiple sectors argue the wolf to not be the sole cause of livestock death, accusing dogs to be a problem in a number of cases^30,32,33^. In the case of England an approximation of 20,000 yearly attacks have been recorded annually (Supplementary Note S1). In these same areas, evidence exists to argue the wolf to preferably attack wild animals over domestic livestock^16–19^. In response to this, the hereby proposed methodology may prove to be an important development in protecting wolf populations from this generated tension. This is especially evident when determining the agent responsible for animal death cannot be confronted using typical approaches^34–36^ thanks to the intervention of other agents such as vultures. This study hereby presents an alternative that can be used to investigate insurance claims concerning livestock predation.

Hybrid artificially intelligent algorithms and morphological data processing provide a starting point for further research into different factors of agricultural and natural science studies. This could consequently reduce the amount of erroneous compensation costs spent by the government in insurance claims of this type. Additionally, while structured light surface scanning was chosen for this study, a further range of other high resolution techniques are also available, including microphotogrammetry and microscopy^38–41^. Coupled with the statistical techniques employed here, investigation into these type of attacks on livestock can now employ a new methodological approach for carcass analysis.

The methods and results presented here are additionally the first to study the different types of canid carnivores in depth. Here we have obtained higher accuracy and classification rates than obtained in some previous efforts to differentiate between other types of carnivores, including jaguars, hyenas and lions, through morphological analyses of their pits^42,43^ and scores^43,44^. These high classification rates have been obtained using a new line of investigation into morphological studies, showing the potential of Artificially Intelligent Machine and Deep Learning algorithms for the processing of such data sets^43^. These methods are able to overcome more traditional techniques into carnivore studies at a much higher resolution.

Nevertheless, it is important to point out that this is not yet the absolute solution to all analytical problems in related fields. These valuable results should be interpreted as an important advance that should guide future experimentation and investigation in order to expand our experimental samples and develop our understanding of different carnivore feeding habits. Similarly, larger sample sizes could be key to building highly robust computational models that can obtain even better results.

From one perspective, investigation is needed to confront whether the size of prey be a conditioning factor in tooth mark morphology, as has been observed in the case of other types of carnivore produced damage^42–44^. While previous efforts regarding other types of archaeological bone surface modifications have argued the size of the animal to not be a conditioning factor in mark morphology^45^, this question is still to be confronted in the case of carnivore tooth marks. If variances were to be observed, the data provided in this study would be applicable only to wolf and dog prey of a large size (including bovids and equids), while further experimental reference collections would be needed for other animal sizes.

Moreover, the inclusion of more carnivore samples can be considered of great importance to resolve similar conflicts in other areas of the world where wolves are not the only predator. In areas such as Southern America, Asia and Africa, predators such as the cougar, jaguar^10,11,25,34,46^, leopard^47,48^ and tiger^49,50^ also require investigating. In the case of Europe we should additionally include the bear^24^. It may thus be considered necessary that research of this type be developed further. Through this, we may be able to provide an additional means of investigating numerous questions regarding agricultural and environmental sciences with a greater ecological, economic and social impact.

## Material and Methods

A total of 83 carnivore tooth pits and 105 tooth scores were studied and compared for this current sample. All marks were collected on long bones of tibiae and radii, considering how diaphysis are denser than epiphyses and are thus more likely to survive during carnivore feeding.

The samples included tooth marks generated by foxes (pits = 29, scores = 41), wolves (pits = 24, scores = 30) and a typical breed of gundog; the Irish Setter (pits = 30, scores = 34). Tooth marks produced by wolves (pits = 24) were obtained from horses of the Cabárceno Natural Park, Cantabria^51^, as well as natural wolf sites in mount Campelo, near Sobrado Dos Montxes, Galicia (scores = 30)^52^. Tooth marks produced by foxes were obtained on samples originating from sheep of Ayllón, Segovia^53^. Dog sampes were obtained on cow bones from Madrid.

Digital reconstructions of different carnivore tooth marks were performed using the David SLS-2 Structured Light Laser Scanner, located in the TIDOP laboratory at the University of Salamanca (Spain). The digital reconstruction protocol^38^ employed the use of a DAVID USB CMOS Monochrome camera, an ACER K132 projector, and a calibration marker board. The scanning process produced 3D models in less than a minute, producing a density of up to 1.2 million points. Final models correspond to meshes that are produced using an algorithm based on the Delaunay triangulation strategy^38^.

Virtual reconstructions of each mark were then imported into different software for landmarking. The Global Mapper software was used to extract cross sections from scores, using tspDig2 (v.2.1.7) for the collection of landmark data. The Avizo software was used for processing entire tooth pits in 3D. Two different landmark models were employed, using a 7-landmark 2D model for scores^44^ and a 17-landmark 3D model for pits^42^. Landmark coordinates were then used to calculate the seven measurements described by Bello and Soligo^39^, which have been successfully adapted and employed for tooth mark analysis^44^. These calculated measurements consider the thickness, depth and various angles of each groove. Both geometric morphometric models and the seven measurements employed in this study have been visually described in Supplementary Fig. S1. Landmark coordinate data was then extracted and converted into a standardized format to be imported into R for further statistical treatment.

### Data analysis

Metric data was first processed using a Principal Components Analysis (PCA). Measurements were then tested for statistical significance using standard multivariate approaches (MANOVA). Depending on the inter-group variability present within each sample, the MANOVA test employed either a Hotelling-Lawley or Wilk’s Lambda test for homogenous or inhomogeneous samples respectively. These steps were repeated including as well as excluding the variable Opening Angle (OA), thus adjusting for this variables’ weight on the overall results^54^.

For geometric morphometrics, landmark coordinates were first subjected to an orthogonal tangent projection, known as Generalized Procrustes Analysis (GPA), in order to normalize data for further multivariate statistical analyses^55–57^. From this, a PCA is performed, reducing the degree of variance to fewer dimensions in order to provide a more efficient comparison of morphology. Additional to analyses of pure morphology in shape space, form space was investigated after re-scaling the data obtained after Procrustes superimposition through the natural logarithm of Centroid Size. Thin Plate Splines were then calculated to explore morphological variation across the different Principal Component (PC) scores^58^. Additional MANOVA testing was performed to assess the significance of morphological variance. PC scores were then extracted to be used for the training of the Machine Learning model.

Considering its recent success in geometric morphometric applications^43^, a supervised Support Vector Machine (SVM) algorithm was used to train a classification model based on morphological tooth mark data. SVMs map out input vectors into a non-linear high dimensional feature space, using hyperplanes to calculate the degree of separation between samples^59,60^ In order to define said feature space, a *kernel* function was used. This helps to overcome traditional limitations imposed by linearity. The constructed hyperplane can be described as a discriminant classifier decision surface which uses a maximized margin or decision boundary to reduce chances of overfitting. The consequent hyperplane divides the samples into *n* – 1 dimension, where *n* is the number of variables.

Data was bootstrapped 1000x to overcome issues that may be produced by the size of samples. Models were then trained using a 70:30% training/testing split. For geometric morphometrics SVMs were trained on the PC scores, while for biometric data the models were trained directly on the measurements. Considering previous observations on the value of the variable *size* in geometric morphometric analysis of taphonomic traces^43^, each model was constructed using either form or shape data considering the type of tooth mark being analyzed. Following the statistical protocol described by Courtenay *et al*.^43^, shape was used for carnivore tooth score marks while form was used for the processing of pits.

SVMs were then trained using *k*-fold Cross Validation (*k* = 10) in order to ensure the model could efficiently adjust its weights. Model optimization was performed following a standard Machine Learning protocol using back propagation to adjust weights during optimization. The corresponding objective function thus employs Gradient Descent to minimize the loss and control overfitting. Additional hyper parameter optimization included the fine tuning of cost and gamma values, established via a random search loop function programmed in R. This algorithm ran for 50 iterations during the tuning of SVM models, using a random combination of cost and gamma values until finding the optimum setting that can ensure the best separation of samples in feature space. These optimal parameters are then extrapolated and used to construct the final classification model.

Model evaluation followed a standardized Machine Learning and Deep Learning approach, using Kappa, Sensitivity, Specificity and Balanced Accuracy values^60^. These values are presented as numbers between 0 (poor) and 1 (high performing) and are calculated via a confusion matrix, assessing the rate and ratio of misclassification and correct classification results. These metrics are interpreted by considering how low Sensitivity and Specificity values indicate high misclassification rates, while the accepted threshold of the Kappa statistic considers a model performing >0.8 to be powerful^60^. The final criteria used for evaluation employed localized loss calculations which could then be used to generate global loss values calculated via the Mean Squared Error (MSE) equation. This equation considers the local error (*E* = *i* − *x*) comparing the real label (*i*) with the classified label (*x*) from the test set. This is then plugged into an equation for global error calculations, as defined in Eq. (1).

1 $$MSE=\frac{1}{n}\mathop{\sum }\limits_{i=1}^{n}{E}^{2}$$

Finally, the SVM training process was microbenchmarked employing 200 iterations and a mean time value in milliseconds was taken as a final result.

A final R code that can be executed on any type of morphological file is available online at https://github.com/LACourtenay/Support-Vector-Machine-for-Morphological-Analysis. A similar version of this code is also available in this repository for the processing of measurements stored in a csv comma delimited file format.

## Supplementary information

Supplementary Notes 1

## Data Availability

Figshare datasets for morphologika files and .csv measurement files are available at: 10.6084/m9.figshare.c.4494218. All R code are available online at: https://github.com/LACourtenay/Support-Vector-Machine-for-Morphological-Analysis. Any queries or issues regarding data or code should be directed to L.A. Courtenay (ladc1995@gmail.com).
