# Supplementary material for: The use of canid tooth marks on bone for the identification of livestock predation
Source: Sci Rep. 2019 Nov 8;9:16301. doi: 10.1038/s41598-019-52807-0 (PMC6841930; doi:10.1038/s41598-019-52807-0)
Supplement: Supplementary file 1 — Supplementary Notes 1 [file 41598_2019_52807_MOESM1_ESM.docx]

**Supplementary Notes 1**

**The use of canid tooth marks on bone for the identification of livestock predation**

*José Yravedra, Miguel Ángel Maté-González, Lloyd A. Courtenay, Diego González-Aguilera, Maximiliano Fernández Deza.*

Correspondence to: [joyravedra@hotmail.com](mailto:joyravedra@hotmail.com)

**This PDF file includes:**

**Supplementary Figure S1 – Page 1**

**Supplementary Notes S1 – Page 2**

**Supplementary Notes S2 – Page 4**

**Supplementary Figure S1 – Geometric Morphometric Landmark Models.** A presentation of the landmark models and measurements taken for each tooth mark. (A) The location of the 7 landmarks for tooth score cross section analysis. (B) The measurements taken for each tooth score cross section. (C) The location of the 17 landmarks for tooth pit analysis.


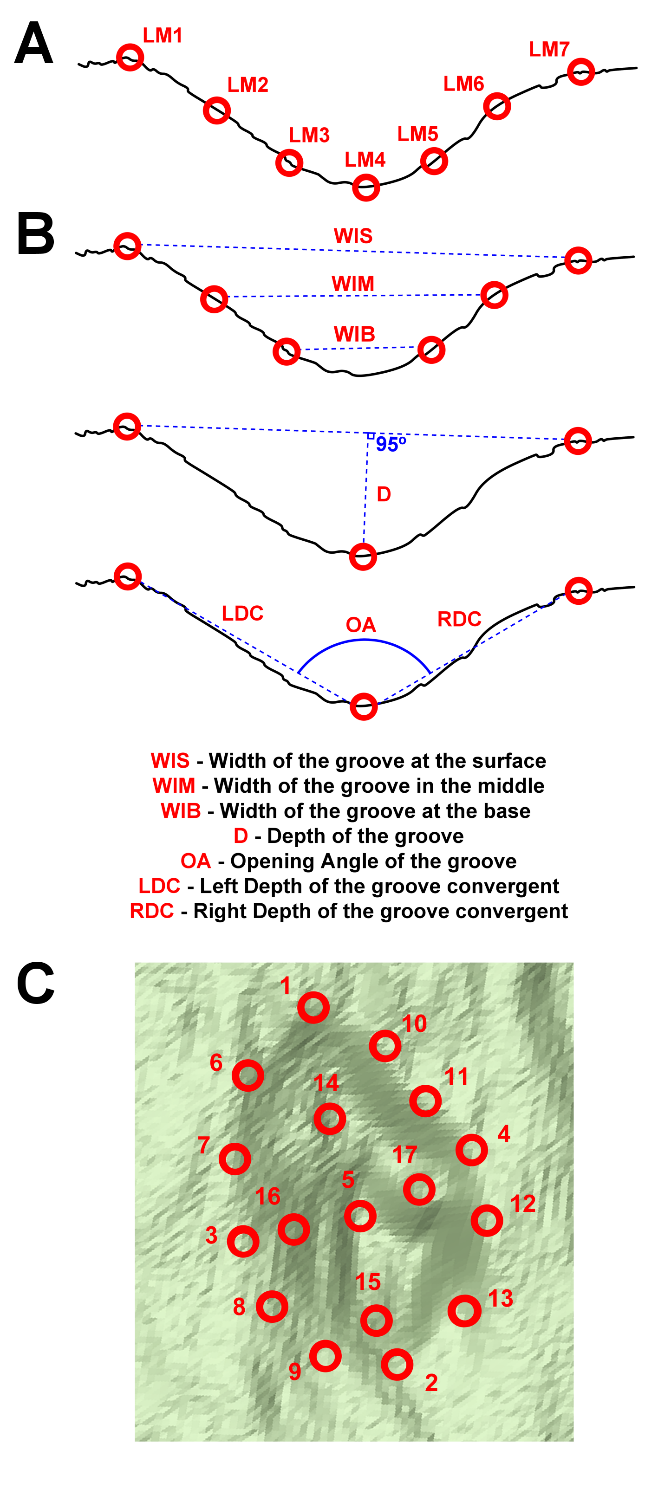


**Supplementary Notes S1:** Conflict generated by wolves and dogs attacks.

**Social Conflict of Wolves**

The wolf is a frequent topic of conversation in recent European and North American media. To cite some of the North American examples, some of the first references of this topic can be cited from the *New York Times*, by Leslie Kaufman, titled “*After Years of Conflict, a New Dynamic in Wolf Country*”, follow by Lance Richardson’s article in *The Guardian* on the 16^th^ of November, 2013, titled “*Who’s Afraid of the American Gray Wolf?*”. Recently, Elizabeth Dunbar published in *Capitol New* a report on state politics and governmental policy titled *“Dead Moose, Puppies Not Enough: House Bans Recreational Wolf Hunt*” on the 30^th^ of April, 2019.

Debates regarding the conservation of wolves is often a question that interests society, especially in European or American countries, even more so in the USA. In support of wolf conservation, supporters of these programs do not usually argue from a simple conservational factor in order to save the species, yet also propose ecological and environmental questions, as seen in the famous Yellowstone park in the USA (e.g. https://www.youtube.com/watch?v=oFV1U8IjiNY). Nevertheless, those against these conservational plans provide the counterargument that protecting these carnivores is counterproductive regarding the security of their livestock, thus having a negative impact on their day-to-day lives and local economies.

Some believe that ranchers and farmers should implement more advanced security methods in order to protect their assets. Examples of this could be seen in the training of dogs such as the popular Mastiff breed to protect their animals, improving installations and enclosures where animals are kept and keeping a better eye on their livestock. Nevertheless, owners of domestic animals argue that they would much rather have fields free of natural predators to avoid further complications.

Regardless of this debate, large amounts of social pressure can still be found in areas of Europe such as Norway, Finland, France and Spain (to name a few), that push towards the elimination of wolf populations for security reasons. In Spain, for example, multiple news articles have been published on this particular topic. On the 14^th^ of September, 2017, Lucía Villa published an article in the *Público* newspaper titled “*Asturias da Vía Libre al Exterminio de los Lobos en un Tercio de su Territorio*” [Translation: *Asturias gives the green light for the elimination of wolves in a third of its territories*]. Similarly, Laura Castro for the *El Comercio* newspaper on the 4^th^ of October, 2017, published “*Los Ganaderos Ayudarán en las Batidas para Dirigir a los Lobos a la Línea de Tiro*” [Translation: *Local Farmers Help in the Herding of Wolves to the Firing Line*]. In the UK, for example, Patrick Burkham published in *The Guardian* an article titled “*It’s Very Scary in the Forst: Should Finland’s Wolves be Culled*?” (26^th^ January, 2018) and later “*Harmless or Vicious Hunter? The Uneasy Return of Europe’s Wolves*” (25^th^ February, 2017), by Patrick Burkham. Similar reports from *The Guardian* by Elisabeth Ulven and Tone Sutterand held the tile “Norway’s Wolf Cull Pits Sheep Farmers against Conservationists” (23^rd^ October, 2016). These issues have reached the extreme of locals taking the issue into their own hands by the unwarented killing wolves, as reported in the *Vanguardia* newspaper of Spain, titled “*La Fiscalía de Asturias Investiga la Muerte de Lobos a Manos de Furtivos*” [Translation: *The Prosecuter’s Office of Asturias Investigates the Death of Wolves at the Hands of Poachers*] (Unknown Author, 20^th^ March, 2017).

From a social standpoint, the wolf can be seen to have created a fair amount of controversy, with a greater social divide in highly agricultural areas. In Germany, for example, the press have responded with enthusiasm to the reintroduction of wolf populations after their disappearance. Gretche Vogel on the 16^th^ of November, 2001, published in *Science* an article titled “*Wolves reappear in Germany*” and Rebeca Pates on the 27^th^ of November, 2018, published on the BBC “*The wolves are coming back*”, expressing similar enthusiasm. Other examples can be seen in the Guardian’s article “*From the Steppe to Central Spain, Europe Echoes to the Howl of the Wolf*” by John Vidal (4^th^ January, 2014). Time after this reintroduction, however, the tone of media coverage on the topic changed, with more publications such as “*Wolf Attacks on Livestock Rise in Germany*” (Timothy Jones, 16^th^ February, 2019, in *Made for Minds*) and “*Farmers Stage Grim Protests against the Big Bad Wolves in Hanover*” (Alexander Johnstone, 15^th^ December, 2016, in *The Local*).

Similar cases have been seen in Denmark, with enthusiastic articles in 2017, such as Patrick Barkham’s post in the Guardian “*Denmark gets its First Wilf Wolf Pack in 200 Years*” (4^th^ May, 2017) followed by tension as shown in “*25 Sheep Killed in Denmark’s Biggest Wolf Attack*” (3^rd^ April, 2019, Ritzau, *The Local*).

**Dog Attacks on Livestock**

Cases of average dogs as predators for livestock have been noted on multiple accounts, with reports of 20,000 attacks on domestic animals by dogs in England alone. This is twice the number of wolf attacks reported in the entire of France (Citation^33^: pg. 51). Aware of these issues, multiple authors have reported cases of insurance fraud, costing the government over 200,000 Euros, product of these incorrect diagnostics for cause of death.

Other examples of domestic animal attacks by dogs can be found in the local media, including BBC news announcements such as “*Dog Attacks on Livestock ‘Devastate’ Farmers*” (28^th^ November, 2017) and “*Dangerous Dogs Act calls after Attack on Sheep Soar”* (8^th^ of April, 2019). Similarly, ITV reported on the 25^th^ of February, 2019, that “*Dog Attacks Cost Farmers Ten’s of Thousands a Year*”. The Nolo law firm and publishing house, for example, also offer advice specifically designated to cases of livestock death product of dog hunting, in “*When Dogs Hunt or Chase Livestock*”, by E.A. Gjelten (https://www.nolo.com/legal-encyclopedia/free-books/dog-book/chapter11-12.html). Other data regarding this problem has been published in magazines such as *Countryfile*, by Mark Rowe, in “*Dog Attacks on Livestock: How Bad is the Problem and What are the Possible Solutions?*”.

In Spain, for example, the newspaper *El Diario* declared a need to confront the problem of dogs in the Basque Country, in the article “*No Podemos Seguir Así. Los Perros Acaban con Nuestro Ganado*” [Translation: *We cannot continue like this. The dogs are ruining our livestock”*] by Joxebi Ramos, 7^th^ November, 2018. Similar reports have been made by Leire Pérez Orizco in *El Correo*, 5^th^ January, 2019, in their article “*Alertan del Aumento de Ataques de Perros a Ovejas en los Montes de Orozko*” [Translation: *Warnings for the Increase in Dog Attacks on Sheep in the Orozko Mountains*]. The state of Navarra, for example, has been declaring the damages produced by dogs for years, as seen in numerous reports from the *Noticias de Navarra* newspaper since the 7^th^ of January, 2007 (https://www.noticiasdenavarra.com/2019/01/07/sociedad/navarra/perros-han-realizado-25-ataques-a-ganado-en-navarra-desde-2007).

Cases have reported abandoned dogs to be an issue in certain areas, with reports by the newspaper *Público* declaring dogs to be a bigger problem than wolves in the 5^th^ of July, 2018 article “*Los Perros Asilvestrados Están Matando tanto o Más que el Lobo*” [Translation: *Abandoned Dogs are Killing more than the Wolf*]. Similar cases have been acknowledged by *El Diario*, with the article “*Los Perros Abandonados Matan en Aragón Más Ganado que el Lobo y el Oso*” [Translation: *Abandoned Dogs Kill More Livestock in Aragón than both the Wolf and the Bear*] (Eduardo Bayona, 12^th^ September, 2018), stating abandoned dogs to be more problematic and costly than even bears. In Formentera, Ibiza, dog attacks have been noted in public media as seen in articles such as “*Denuncian Nuevos Ataques de Perros a Ganado en Formentera*” [Translation: *Complaints about New Attacks of Dogs on Livestock Reported in Formentera*].

In other areas of Europe, similar cases have been seen in countries such as France, with the report of *“Pyrénées. Les Chiens Tuent Plus que les Ours*” [Translation: *Pyrenees. Dogs Kill more than Bears*] (ladepeche.fr, 21^st^ August, 2011) and “*Ses Chiens Avaient Attaqué des Moutons, le Propriétaire Condamn*” [Translation: *The Condemned Owner of Sheep Attacked by Dogs*] (29^th^ September, 2013).

**Supplementary Notes S2: Subsidies Derived from Compensation to Farmers**

Consequence of recent media activity, social conflict and different ecological issues, different government plans have been proposed to overcome these issues with staggering financial schemes. One possible case study, for examples, can be seen in the US Government’s payment of 800,000 US Dollars in compensation of Katie Willies (physe.org. Alberta, 17^th^ January, 2018), as documented in the article “*Reimbursing Ranchers for Livestock Killed by Predators Supports Conservation Efforts”.* In Europe, similar cases have been observed regarding different compensation subsidies, such as the case published by Paola Tamma (euroactive.com, 17^th^ January, 2018) with the article titled “*Are We Crying Wolf? A European Tale of Farmers vs Nature*”, stating that 10,000 victims of wolf predation have cost the French Government over 3 million Euros. In Italy, costs have risen over 1.5 million Euros, while in Spain, 1.6 million Euros have been spent on this case between the years 2014 and 2016 (Citation^33^: pg. 70).

In Spanish states such as Asturias, an estimate of 1 million Euros are being spent a year, surpassing the 11 million Euros spent between the years 2000 and 2015, as published by www.senderismoenasturias.es in 2016 with the article “*El Lobo/Tsubu/Llubu (Canis lupus signatus) en Asturias*” [Translation: *The Wolf/Tsubu/Lubu (Canis lupus signatus) in Asturias*]. In the state of Cantabria, compensation costs are lower however are still surmountable to 243 thousand Euros, according to the reports of Nacho García Ucelay in the *Diario Montañes* newspaper on the 14^th^ of June, 2018, with the article titles “*Los Daños Causados por la Fauna Salvaje le Cuestan al Gobierno de Cantabria 470.000 Euros*” [Translation: *The Cost of Damages Produced by Wild Fauna have Cost the Cantabrian Government 470,000 Euros*]. The state of Madrid, on the other hand, has recently been reported to be increasing their budget from 60 to 120 thousand Euros for livestock predation compensation, as reported in *The Guardian* by Sam Jones with his article titled “*Madrid to Double Farmers’ Compensation Fund for Wolf Attacks*” (28^th^ November, 2016).

One particular case study can be seen in the Spanish state of Castilla y León, where a cost of 130,000 Euros was paid out for claims of wolf predation, as published by the *El Adelantado de Segovia* newspaper, in “*Dos Sentencias Condenan a Pagar 130.000 Euros por Ataques de lobos”* [Translation: *Two Sentences Cost the Town Hall 130,000 Euros for Attacks by Wolves*] (Guillermo Herero, 8^th^ November, 2015).

It is necessary to note alongside these reported costs those produced by incorrect pay outs, product of errors when interpreting the cause of death for some livestock. One account has been noted of 200,000 Euros being spent on claims of wolf attacks that were actually product of dogs^25^. Similar estimations have predicted governments to have handed out large amounts of money product of inadequate means of determining the agent responsible for killing their livestock.

In other parts of Europe, Germany and Switzerland have also invested large amounts of money in the preparation of protection plans orientated towards the protection of livestock with a cost of over half a million Euros. Such instances have been reported by newspapers such as *The Local* in “*Farmers Stage Grim Protests against Big Wolves in Hanover*” (Alexander Johnstone, 15^th^ December, 2016) as well as cases in the United Stated where Katie Willies reports “*Reimbursing Ranchers for Livestock Killed by Predators Supports Conservation Efforts*” (physe.org, Alberta, 17^th^ January, 2018). Needless to say, the data collected and presented here is not exclusive to the 21^st^ century, as seen in data provided by Fritts^3^ and Fourli^23^ describing the increase in costs and annual subsidies across the EU and North America.

Finally, in summary, the following table describes and presents a collection of figures describing cases where large amounts of money have been sent covering the costs of damage produced by livestock predation:

| Country | Quantity | Source |
| --- | --- | --- |
| France | 3 mill. € | Paola Tamma (euroactive.com, 17^th^ January, 2018) |
| Italy | 1.5 mill. € | Paola Tamma (euroactive.com, 17^th^ January, 2018) |
| Spain (Asturias) | 11 mill. € (year 2000-2015) | www.senderismoenasturias.es |
| Spain (Cantabria) | 470 000 € | Nacho García Ucelay in the *Diario Montañes* newspaper on the 14^th^ of June, 2018 |
| Spain (Madrid) | 60-120,000 € | *Madrid to Double Farmers’ Compensation Fund for Wolf Attacks*” (28^th^ November, 2016). |
| Spain (Castilla León) | 130,000€ | *El Adelantado de Segovia* newspaper, in “*Dos Sentencias Condenan a Pagar 130.000 Euros por Ataques de lobos* |
